# Triboelectric-induced ion mobility for artificial intelligence-enhanced mid-infrared gas spectroscopy

**DOI:** 10.1038/s41467-023-38200-6

**Published:** 2023-05-02

**Authors:** Jianxiong Zhu, Shanling Ji, Zhihao Ren, Wenyu Wu, Zhihao Zhang, Zhonghua Ni, Lei Liu, Zhisheng Zhang, Aiguo Song, Chengkuo Lee

**Affiliations:** 1 School of Mechanical Engineering, Southeast University, Nanjing, 211189 P. R. China; 2 Department of Electrical and Computer Engineering, National University of Singapore, Singapore, 117576 Singapore; 3 Center for Intelligent Sensors and MEMS (CISM), National University of Singapore, Singapore, 117576 Singapore; 4 NUS Suzhou Research Institute (NUSRI), Suzhou, 215123 P. R. China; 5 School of Instrument Science and Engineering, Southeast University, Nanjing, 211189 P. R. China

**Keywords:** Infrared spectroscopy, Environmental monitoring, Sensors and biosensors

## Abstract

Isopropyl alcohol molecules, as a biomarker for anti-virus diagnosis, play a significant role in the area of environmental safety and healthcare relating volatile organic compounds. However, conventional gas molecule detection exhibits dramatic drawbacks, like the strict working conditions of ion mobility methodology and weak light-matter interaction of mid-infrared spectroscopy, yielding limited response of targeted molecules. We propose a synergistic methodology of artificial intelligence-enhanced ion mobility and mid-infrared spectroscopy, leveraging the complementary features from the sensing signal in different dimensions to reach superior accuracy for isopropyl alcohol identification. We pull in “cold” plasma discharge from triboelectric generator which improves the mid-infrared spectroscopic response of isopropyl alcohol with good regression prediction. Moreover, this synergistic methodology achieves ~99.08% accuracy for a precise gas concentration prediction, even with interferences of different carbon-based gases. The synergistic methodology of artificial intelligence-enhanced system creates mechanism of accurate gas sensing for mixture and regression prediction in healthcare.

## Abstract (web summary)

Isopropyl alcohol can play a significant role in anti-virus diagnosis but generally yields limited response in conventional detection. Here, authors propose a methodology of AI-enhanced ion mobility and mid-infrared spectroscopy for accurate gas identification despite presence different carbon-based gases.

## Introduction

The need for health and safety has been increasing the demand for isopropyl alcohol (IPA) as a vital element of anti-virus hand sanitizers^1–5^. Many virus-perishing methods are investigated for combatting pandemic or cold diseases, such as managing indoor air, increasing gas ventilation, optimizing humidity levels, maximizing air filtration efficiency, and the widespread use of IPA. IPA is a flammable and colorless liquid with a fruity odor and a slightly bitter taste. It is used to clean electronic components and to remove the thermal paste from integrated circuit chip packages. Due to its extensive use, various healthcare problems may occur along with the exposure or inhalation of the IPA molecules, e.g., skin irritation, nervous system illness, and respiratory damage. As a result, rapid and accurate detection of IPA molecules becomes critical. The methods for IPA sensing include chemiresistive, gas absorption-based, two-dimensional(2-D) material-based, ion mobility, electrochemical, and other sensors^6–16^. However, such IPA sensors can only measure the total gas concentration among various volatile organic compounds(VOCs), and they have limitations such as slow response, poor selectivity, and insufficient accuracy. Thus, a sensing system with fast response, good selectivity, anti-interference, and high sensitivity for IPA identification is critical.

Ion mobility and mid-infrared spectroscopy are two well-known analytical techniques for identifying of gas-phase compounds. Scientists focus on ion mobility and investigated many approaches to increase sensing ability, e.g., producing ions by β-radiation, ultraviolet (UV) irradiation, and others. However, this approach requires a high-voltage source and a strict operating environment (e.g., vacuum or low pressure), yielding expensive and bulky equipment^17–23^. Mid-infrared spectroscopy is a non-destructive method applied to identify the vibrational modes of gas molecules, and it has widespread applications in environmental monitoring, pharmacy, chemical analysis, bio-sensing, and the pharmaceutical industry. Regarding the sensitivity enhancement in mid-infrared spectroscopy, it is found that the instantaneous strong electrostatic field could be further tailored to the molecular vibration along with the optics refection response, thereby enhancing the absorption effect of infrared spectroscopy. In short, conventional ion mobility sensing has the constraint of the requirement of a strict measurement environment, whereas conventional mid-infrared spectroscopy is limited by poor response during low-concentration identification. Overall, both methods have drawbacks in IPA sensing at this point.

Herein, three critical issues need to be addressed in order to improve the response of IPA molecules using existing ion mobility or mid-infrared spectroscopy. Firstly, focusing on the interaction of molecules with infrared irradiation, high-voltage plasma induces a strong electromagnetic field, yielding a coupling effect, which amplifies the mid-infrared absorption response. Therefore, plasma-enhanced infrared absorption spectroscopy enables the ultrasensitive detection of molecules with the aid of an existing high-voltage power source (ion mobility). Secondly, regarding ion mobility, a triboelectric nanogenerator^24–33^ was discovered in 2012, allowing for an additional high-voltage output from mechanical vibrations. With the aid of triboelectric, Wang et al. reported respiration health based on AI-assisted diagnosis by multi-self-calibrated parameters from TENG with accuracy >95.21%^33^. However, anti-virus VOCs diagnosis from hybrid mechanism (mid-infrared spectroscopy) and data-enhanced algorithms still need to be investigated. For example, the ion power source from the triboelectric nanogenerator could be greatly optimized and provide ion mobility in cold environments and ambient air pressure. Thirdly, artificial intelligence (AI) is a hot research topic dramatically promoted with the fifth-generation cellular network technology (5 G). A cloud server would easily handle plenty of data based on deep learning algorithms in a short time^34–39^. Providing feasible big data processing using machine learning and deep learning techniques (t-distributed stochastic neighbor embedding (t-SNE), linear discriminant analysis (LDA), principal component analysis (PCA), synthetic minority oversampling technique (SMOTE), and deep neural networks (DNN) regression), which may reinforce the characteristic IPA data-sets from ion mobility and mid-infrared. Thus, the AI-enhanced methodology would be an ideal solution for IPA detection in factories or personal healthcare, where identifying IPA molecules requires rapid response, high accuracy, good selectivity, anti-interference, and high sensitivity.

To realize the IPA detection with good selectivity, fast response, and high sensitivity, we propose an artificial intelligence (AI)-enhanced chemical detection based on IMMS assisted by a multi-switched triboelectric nanogenerator. It provides an additional high-voltage power source for ion mobility and plasma enhancement for mid-infrared sensing. The plasma-enhanced mid-infrared response in IPA sensing exhibits a good concentration prediction with ~0.87 *R*^*2*^ score by DNN regression, which can be improved to ~0.98 *R*^*2*^ score using SMOTE before DNN regression. What’s more, it is also demonstrated that the data preprocessing method could remove the background calibration with high accuracy in feature classification. In the perspective of sensing fusion between IMMS, the regression performance in concentration prediction can be enhanced to a ~0.99 *R*^*2*^ score. Moreover, even in the case of different gas interferences, this synergistic methodology of IMMS reaches ~99.08% accuracy in our study using the AI-enhanced method and high-voltage triboelectric cold ion mobility.

## Results

### AI-enhanced detection based on IMMS

**Fig. 1:**
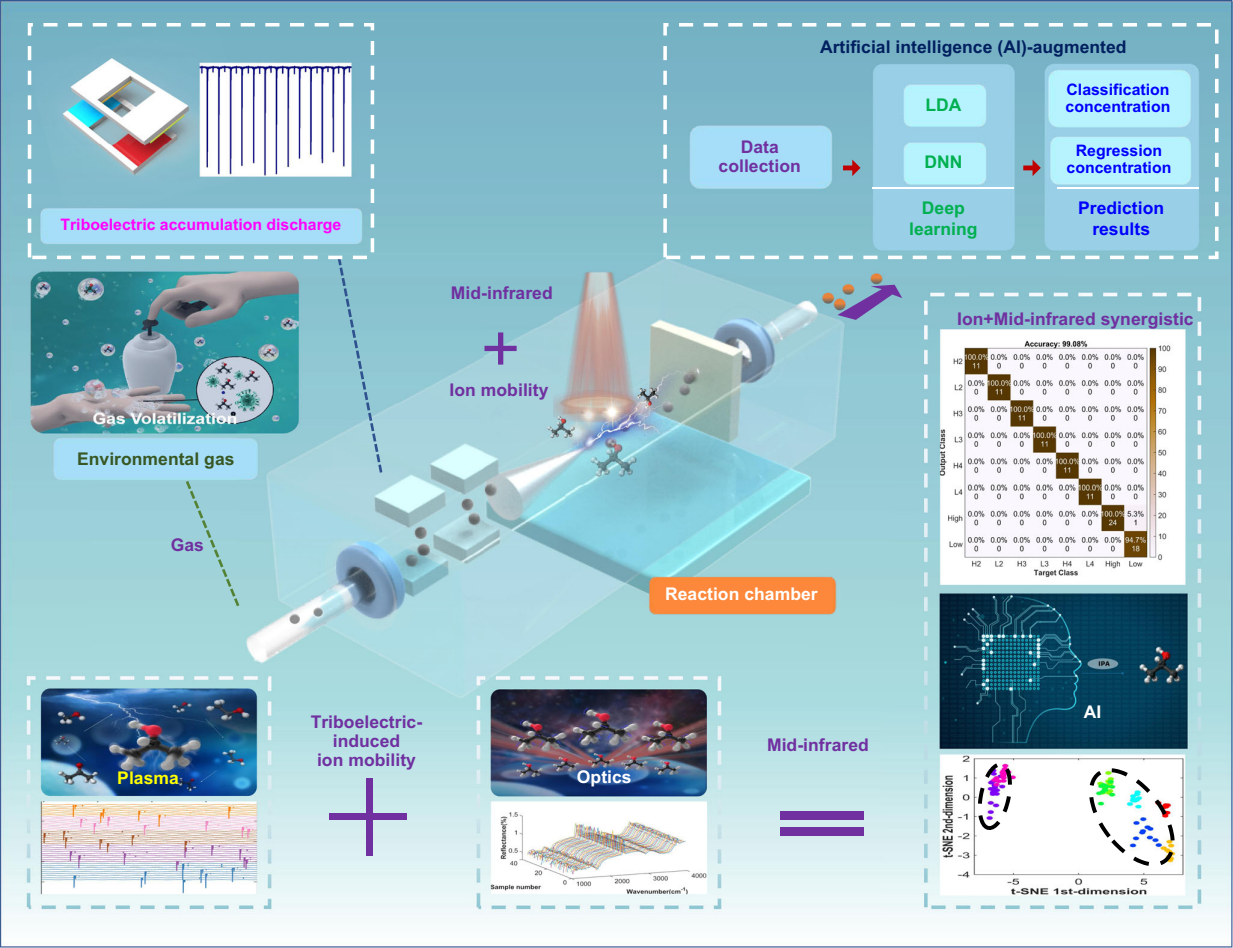
The schematic diagram of AI-enhanced chemical sensing from synergy methodology by IMMS. Enhanced mid-infrared gas spectroscopy, the schematic diagram of artificial intelligence(AI)-enhanced chemical sensing from synergy methodology by ion mobility and mid-infrared spectroscopy, the concept of triboelectric-induced ion mobility and mid-infrared with synergistic by AI.

As shown in Fig. 1, IPA, as one of the important elements of hand sanitizers, has recently been globally recommended and used by doctors and the entire population. Long-term exposure to the IPA environment may induce serious healthcare problems in humans, e.g., headache, skin irritation, or nervous system illness. The accurate detection of IPA species is an urgent need to provide a safe environment. The most common method for accurate detection of IPA is based on ion-mobility or mid-infrared. However, the detection is restricted to specific conditions (low air pressure and high temperature). To address this issue, we present a self-powered triboelectric generator as an additional high-voltage source resulting in cold ion plasma at ambient air pressure. The use of ion mobility in accurate IPA detection under low and ambient air pressures is greatly enhanced by the high-voltage generation from the triboelectric nanogenerator based on mechanical vibrations. Meanwhile, mid-infrared spectroscopy reduces the response of IPA molecules along with absorption and reflection, which wavelength and response are accurately achieved. The synergistic methodology of IMMS for chemical sensing becomes a solution for the fast and accurate detection of a gas mixture at low concentrations taking advantage of the triboelectric nanogenerator. Herein, a multi-switched manipulation triboelectric nanogenerator is developed to reach a high-voltage output without any external battery with the theory of charge accumulation. The mechanics of the high-voltage generator can be found in Fig. S1. The surface interface effect of the triboelectric and the transfer of charges, cause the multi-switches alternate between “on” and “off” states, leading to a continuous accumulation of charge that generates an extra high voltage from the ground. The Bennet doubler was proposed nearly 100 years ago. It can be achieved by an in-plane mechanical structure of the multi-switched manipulation triboelectric nanogenerator based on the back-and-forth sliding. Thus, the generated charge from the ground would be accumulated on the material’s surface using the electrostatic effect. The open-circuit voltage (*V*_oc_) and the accumulative open-circuit charges (*Q*_oc_) are presented in Figs. S2, S3 in the supplementary information.

The IPA response mechanism is from the characterizations in absorption and reflection by mid-IR. The external-provided strong electric field dramatically improves the sensitivity of the IPA detection due to the high-voltage fluctuation from the plasma discharge onto the vibrational mode of the molecule structure (coupling effect). Thus, the synergistic mechanism of ion-mobility sensing and enhanced-mid-IR reflection enables chemical sensing to achieve fast response times and accurate detection (two kinds of data in computing). Furthermore, the data processing with an AI-enhanced approach would combine the advantage of both ion-mobility sensing and enhanced-mid-IR response resulting in extra high accuracy and wide-range detection. The detailed experiment photos of the AI-enhanced IMMS can be found in Fig. S4. The AI algorithms are conducted to identify the accuracy of IPA concentrations, e.g., t-SNE, LDA, PCA, SMOTE, and DNN. Through the interaction of IPA molecules with plasma discharge and mid-infrared irradiation, the species and concentration of IPA can be accurately identified along with the output signal response. The raw data obtained from both mechanisms is then sent to a computer for further analysis. Assisted by the deep learning technology, the information from the synergistic mechanism of ion discharge and mid-infrared methodology can be identified with an accuracy of ~99%, whereas the conventional recognition of IPA can only reach an accuracy of ~50%^37^. Thus, the AI-enhanced approach can process multi-modal data from the collected data, and it can be further improved with the synergistic methodology to realize the fast response, wide detection range, and accurate detection of a gas mixture.

### Ion mobility analyzer for gas identification

**Fig. 2:**
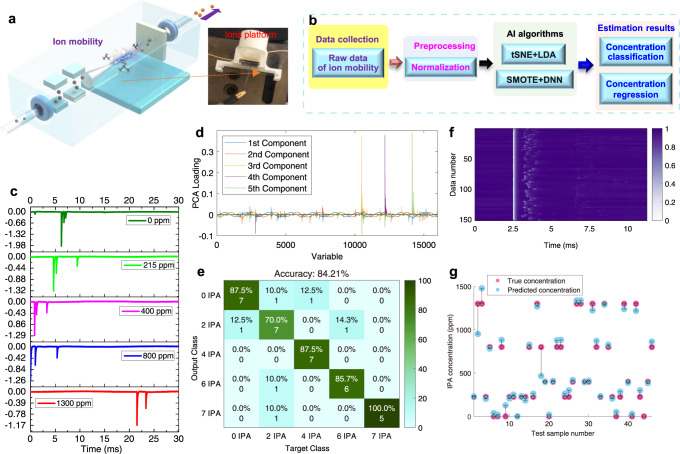
IPA detection using ions mobility analyzer. **a** Ion mobility platform. **b** The schematic diagram of the deep learning method. **c** The dark current pattern of the different concentrations IPA, 1300 ppm, 800 ppm, 400 ppm, and 215 ppm, respectively. **d** PCA loading classification. **e** tSNE+LDA classification accuracy of different concentrations. **f** IPA concentration with SMOTE in data map. **g** The concentration estimation results after SMOTE + DNN.

The ion mobility system is constructed in a tip-plate electrode configuration, high-voltage component, and current collector, as shown in Fig. 2a. The tip-plate electrode configuration provides a site for ion mobility and various gas environments, and the current collector is used to collect dark current from ion mobility for monitoring. The high-voltage component is a triboelectric nanogenerator that could generate high voltage from mechanical vibrations. The voltage range from multi-switched manipulation triboelectric nanogenerator can reach up to ~2700 V under the load resistance 800 MΩ (Fig. S5) based on our observation. Figure 2b depicts the schematic diagram of the AI-enhanced ion mobility methodology. Through the use of the deep learning methods, both the estimated value of IPA concentrations can be accurately determined. The deep learning method in our calculation contains tSNE+LDA and SMOTE + DNN, which are adopted for classification and regression to assist the IPA identification. This method is chosen based on the curve pattern of the signals. Figure 2c and Figs. S6, 7 in the supplementary information, demonstrate the patterns exhibited by the raw data. The data for specific IPA concentrations including 1300, 800, 400, 215 ppm, each of which exhibits a clear pattern under different concentrations. The reasons to choose those concentrations are both the limit of the calibration sensor (SKY2000-VOC) and the range of the IPA in the air from hand sanitizer which is enough to show the air quality status. The picked concentrations (i.e., 1300, 800, 400, 215, and 0 ppm) are the reference to evaluate the sensing performance of AI-enhanced IMMS (wide range and response sensitivity). The t-SNE feature dimension reduction or PCA dimension reduction with different concentrations is shown in Fig. 2d and Fig. S8 in the supplementary information, and classification results using t-SNE + LDA are given in Fig. 2e. The ion mobility pattern represents the concentrations of IPA molecules. The recognition accuracy of different concentrations can reach ~84.21%, according to our observation. Therein, the accuracy is calculated as follows:

1 $${{{{{\rm{Accuracy}}}}}}=\frac{{Number}\,{of}\,{correctly}\,{outputs}}{{Total}\,{sample}\,{number}}\times 100\%$$

In order to enhance the accuracy of IPA detection, we adopted raw data analysis both with and without SMOTE + DNN regression for an in-depth study of the dark current pattern, as illustrated in Fig. 2f and Figs. S9, 10 of the supplementary information. It is observed that the SMOTE + DNN regression gives clear accuracy improvement from Fig. 2g and Fig. S9b. The detailed modeling process can be found in Method Section. Use ${y}_{i}$ to represent the actual concentration, ${f}_{i}$ to represent the predicted concentration, and $\hat{y}$ to represent the average of the actual concentration. The computational formula of the *R*^*2*^ score is as follows:

2 $${{{{{{\rm{R}}}}}}}^{2}=1-\mathop{\sum}\limits_{i}{\left({y}_{i}-{f}_{i}\right)}^{2}/\mathop{\sum}\limits_{i}{\left({y}_{i}-\hat{y}\right)}^{2}$$

Here the *R*^*2*^ score for the test dataset was 0.96, which represents a good regression performance.

### Mid-infrared enhancement

**Fig. 3:**
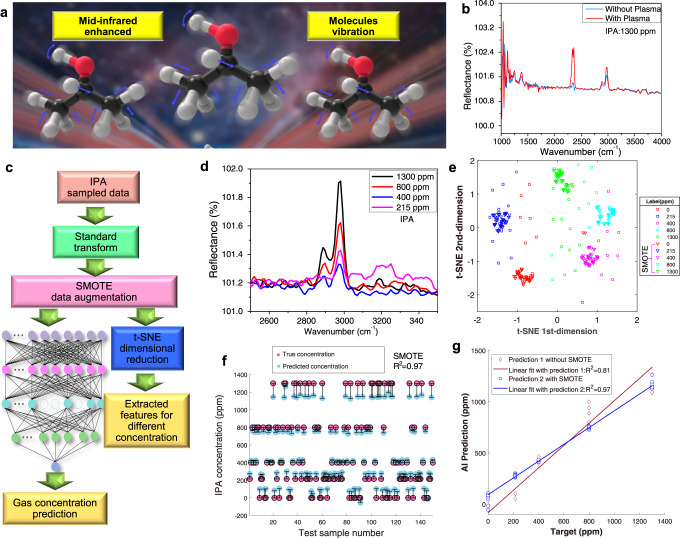
Mid-infrared enhancement plasma discharge to different concentrations of IPA. **a** Schematic diagram of enhancement mid-infrared for IPA species. **b** With/without plasma mid-infrared response in IPA. **c** The schematic diagram of the deep-learning. **d** The response of the mid-infrared of different concentrations in IPA. **e** t-SNE features without and with SMOTE augmentation. **f** Results with SMOTE + DNN augmentation. **g** SMOTE + DNN augmentation with different values of *R*^*2*^.

Figure 3a illustrates the plasma-enhanced vibrational spectroscopy of IPA molecules in the mid-infrared spectral region which significantly enhances their sensitivity. The IPA response over time in the mid-infrared spectral region is shown in Fig. 3b and Fig. S11 in the supplementary information, and is used to illustrate the difference in measurement data depending on ion mobility and to understand its influence during the mid-infrared response. At a wavenumber of ~2400 cm^−1^, characteristic for CO_2_, a ~1–3 times higher response is observed than without plasma (the red curve and the light blue curve, respectively). The AI-enhanced methodology for mid-infrared spectroscopy is shown in Fig. 3c to describe the IPA processing data. The data processing contains the standard calibration, SMOTE-enhanced method, and t-SNE classification. The SMOTE method is used to predict the IPA concentration. The goal of the SMOTE is used to augment the dataset of the IPA concentration. The t-SNE classification is used to classify the measured IPA molecules, and the DNN with multilayers is used to predict the IPA concentration. Figure 3d and Fig. S12 in the supplementary information show the IPA concentration vs. the mid-infrared response. Figure 3d is the zoon out of Fig. S12 which is easier for readers to observe with the wavenumber of 2500 cm^−1^~3500 cm^−1^. The relationship between different IPA concentrations with almost a linear numerical relationship is shown in Fig. S13 in the supplementary information. With reference to concentrations chosen (i.e., 1300, 800, 400, 215, and 0 ppm), it concludes that the minimum IPA molecule concentration is determined by the FTIR equipment’s resolution and the accuracy of AI engineering. Furthermore, the limit of detection (LOD) is evaluated by the performance of the equipment relating to noise. It can be well calculated by the modified Beer–Lambert equation for the variations in the optical path length and the actual absorption data^39–42^. To accurately evaluate the noise in real measurement, the 0 ppm IPA molecules are chosen to extract the signal fluctuations of these spectra. By plotting the total noise and the output signal with SMOTE + DNN regression together, it can analyze the LOD of the Ai-enhanced IMMS. The SMOTE + DNN regression of the signal above the noise at 0 ppm indicates that the LOD of our IPA could reach a prediction of ~40 ppm based on the estimated mean error using an improved IMMS technique (Fig. S13b). As shown in Fig. 3e and Fig. S14, 15 in the supplementary information, these raw data can be well handled to provide visualization for readers based on the t-SNE classification. The SMOTE augmented dataset of squared markers has more remarkable t-SNE features and more compact clusters compared with triangle markers. It also shows the feasibility of the deep learning algorithm for the identification of IPA molecules. To further clarify the difference in the big range of accuracy identification, the SMOTE + DNN regression prediction method for calculation, as shown in Fig. 3f and Fig. S16 in the supplementary information, shows good prediction at almost every IPA concentration. The regression performance *R*^*2*^ score with SMOTE can be improved from 0.91 to 0.97, whereas the *R*^*2*^ score from 0.87 to 0.98 can be observed in Fig. 3g and Fig. S17 in the supplementary information.

### Robust mid-infrared demonstration from the AI-enhanced approach

**Fig. 4:**
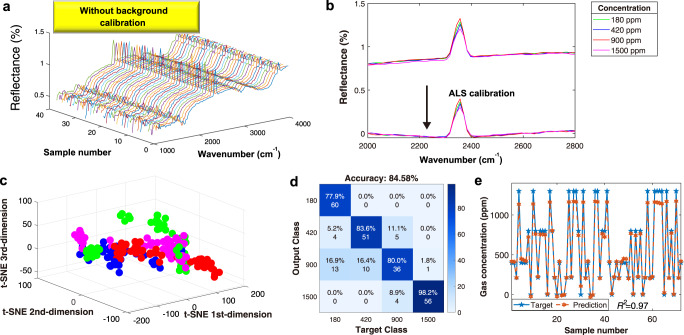
AI-enhanced methodology without any initial background calibration. **a** The schematic diagram of the raw data and deep learning process. **b** The data with ALS calibration. **c** The t-SNE feature extraction and classification. **d** The results by LDA classification. **e** DNN prediction results in different concentrations of IPA.

The background calibration by FTIR equipment is critically important before the real optical measurement. The goal of the background calibration is to provide the correct form of the curve, by removing unnecessary information (environmental perturbation). However, the manual calibration before the real measurement is inconvenient and inefficient. To avoid this manual process (labor-consuming), we adopt an artificial intelligent method to provide the right concentration measurement. The raw data without data preprocessing is shown in Fig. 4a. The data preprocessing can be implemented by the standard transform, SMOTE, and ALS calibration. Afterward, the t-SNE is used for feature extraction. Then LDA and DNN are used to assist in gas classification and prediction. The ALS calibration is shown in Fig. 4b and Fig. S18 in the supplementary information. The obtained curve is uniform in the right baseline. The t-SNE classification and its results shown in Fig. 4c, d conclude that LDA can be adequately classified by all different concentrations of IPA from the mid-infrared data with ~84.58% accuracy. We adopt the DNN prediction method to improve the sensitivity, with *R*^*2*^ score of 0.97, as shown in Fig. 4e and Fig. S19 in the supplementary information. It concludes that all different concentrations could be obtained even without any calibration step in the FTIR system.

### IPA identification demonstration by synergistic methodology in the gas mixture

**Fig. 5:**
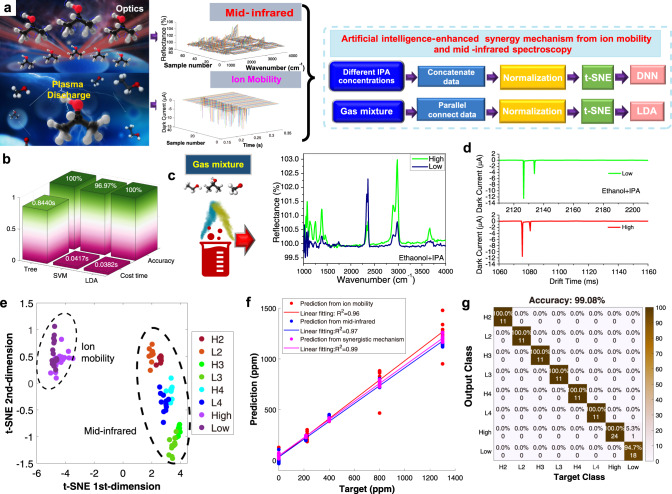
Mixture gas detection based on the synergy of IMMS. **a** The characteristic and the deep learning method. **b** Comparison results among linear discriminant analysis, support vector machine, and decision tree. **c** Mid-infrared raw data with a different mixture IPA. **d** IPA with ethanol disturbance by ion mobility. **e** t-SNE features to all the mixture IPA. **f** AI-enhanced method for IPA gas estimation with ~0.99 *R*^*2*^ score. **g** AI-enhanced method for mixture gas identification with ~99.08% accuracy.

Figure 5 depicts the synergistic methodology of both IMMS. As shown in Fig. 5a, the signals from both are sent to a machine learning tool for calculation. The IPA molecules in the IMMS approaches are dramatically shaken by the super-high voltage. In terms of concentration estimation, data from two methods that measure the same concentration are concatenated, and then the t-SNE is used for feature extraction and DNN for concentration estimation. The data of gas are parallel connected and addressed in such a way that the gas characters are classified using LDA to distinguish different IPA concentrations or different species of the gas. The AI-enhanced sensor-fusion mechanism is implemented for the IPA concentration. The purpose of LDA is to maximize the between-class variance and minimize the within-class variance. For the regularized LDA in MATLAB, it is assumed that all classes have the same covariance matrix and the predictor covariance treatment as follows,

3 $${\hat{\varSigma }}_{\gamma }=(1-\gamma )\hat{\varSigma }+\gamma {{{{{\rm{diag}}}}}}(\hat{\varSigma })$$

where $\hat{\varSigma }$ is the empirical, pooled covariance matrix, and $\gamma$ is the amount of regularization. LDA model is trained using the labeled data from the plasma-enhanced mid-infrared spectral region or ion mobility analyzer. The synergistic methodology of IMMS is represented in Fig. S20 in the supplementary information. The response performance in IPA concentration estimation with three modes can be observed in Table S1. It is observed that the time costs for training in IMMS are the longest, however, it provides a wider concentration detection range, and higher accuracy compared with the single ion-mobility or mid-infrared spectrum method. Comparison results among linear discriminant analysis, support vector machine, and decision tree, demonstrate that the LDA-based AI method achieves the best performance with the least time cost and highest accuracy. To evaluate the effectiveness of proposed AI-enhanced methods in IPA identification, we compare the results with the support machine learning (SVM, PCA, or DNN)^34,36,38^ and a decision tree. As shown in Fig. 5b in the supplementary information, the LDA-based method combined with PCA is most suitable for the AI-enhanced IMMS to recognize gas molecules from mixtures. In the terms of calculation cost time, the LDA-based IPA identification method spends the least amount of time for 0.0382 s, while the SVM-based and tree-based methods spend 0.0417 s and 0.8440 s, respectively. Moreover, the LDA-based and tree-based methods achieve 100% accuracy in the IPA identification from a gas mixture, while the SVM-based method obtains 96.97% accuracy. It concludes that LDA-based AI method achieved the best performance with the least time cost and highest accuracy. In addition, as shown in Figs. S21–S23 in the supplementary information, to verify the validation with different split ratio training to a higher accuracy and a lower estimated error, the ratio settings for train/test split were chosen 70:30, 75:25, and 80:20. It concludes that the result with 80:20 presents best training effect in our AI-IMMS molecule calculation case. However, considering more training samples, the ratio in the formal experiment is 70:30.

IPA as one of the important elements in VOCs family can affect human health and be used for the assessment of various diseases. For example, IPA has been widely used as antiviral hand sanitizer. Also, many studies have indicated that IPA levels can reflect the severity of lung cancer, serving as a specific biomarker for early diagnosis, which deserves attention and further research^43–45^. To further demonstrate the IPA detection in mixture gases, as shown in Fig. 5c and Figs. S24, 25, IPA mixed with one, two, or three different kinds of species appears. A higher IPA concentration in the IPA gas mixture induces a strong peak response at a wavenumber of ~1800 cm^−1^, clearly implying that the peak response of different gas species can be well-identified. The gas species are determined by the response of molecules with their mode at a certain wavenumber, whereas their concentrations are indicated by the response value. IPA mixture gas detection based on the plasma-enhanced mid-infrared spectrum is represented in Fig. S26 in the supplementary information. It is found that the best model by PCA and LDA yields an accuracy of ~100%. Thus, the AI-enhanced methodology demonstrates the identification of the IPA characters. Furthermore, as shown in Fig. 5d, dark current from ion mobility can be represented as a pattern. Fig. S27 demonstrated that the concatenate data has an advantage of data augmentation by the synergistic methodology of IMMS. As shown in Fig. 5e, the results indicate that the data dots cluster is directed to specific concentrations. With the auxiliary sensing fusion techniques and DNN, the concentration estimation reaches a result of ~0.99 *R*^*2*^ score, which is improved compared with a single measurement as illustrated in Fig. 5f. In addition, assisted by the SNE and LDA methods, the classification is shown in Fig. 5g, yielding an accuracy of ~99.08%. Thus, the AI-enhanced methodology demonstrates the identification of the IPA characters and their concentrations.

## Discussion

In conclusion, we propose a synergistic IMMS mechanism for AI-enhanced chemical sensing to achieve rapid response and accurate detection of a gas mixture. A triboelectric nanogenerator for high-voltage plasma is proposed to overcome the environmental pressure constraints to ion mobility and the weak response and reflection detection of gas molecules, limiting the use of mid-infrared spectroscopy. The reported self-powered ion mobility reaches up to almost two times higher accuracy with the aid of AI-augmentation by automatic extracting of the specific features than the conventional method. It is also shown that cold plasma from the triboelectric generator enhances the mid-infrared response for IPA sensing with a good linear prediction by deep learning. Moreover, the data processing could remove the background calibration based on our observation with a labor-consuming effect. Due to the challenges in IPA detection, the AI-enhanced approach is successfully demonstrated by extracting the features from the IMMS data with ~99.08% accuracy. This method can process multi-modal data from collected data, and can be combined with existing methodology to realize the synergistic mechanism.

## Methods

### Fabrication of the multi-switched triboelectric nanogenerator

The multi-switched triboelectric nanogenerator contains the bottom pair of plates and a top movable pair of plates with a contact area of 49 cm^2^. For power output, a positive triboelectric polymethyl methacrylate (PMMA) dielectric is connected with negative fluorinated ethylene propylene (FEP). The laser cutting machine is used to cut the acrylic plate for component assembly. The conductive nickel textile is used to connect and separate with/from an electrode via the action of “on” or “off”.

### Characterization

A programmable electrometer (Keithley model 6514) is adopted for *V*_oc_, and *Q*_oc_ parameters. The infrared spectra are recorded using an FTIR spectrometer (Agilent Cary 610 Series), which operates at wavelengths from 4 to 8 μm. The measurement environment is at room temperature, exhibiting the tolerance of the test environment. The calibration sensor of the IPA is a commercial device.

### IPA platform

The platform is made of the multi-switched triboelectric nanogenerator, the pre-mixture IPA chamber, and the FTIR equipment. The pre-mixed chamber is constructed to mix different gases. The calibration sensor (SKY2000-VOC) is used to identify the specific concentration of IPA. An oscilloscope is used to monitor the dark current from the collection electrode to record the dark current response along with the gases. A computer is used to record FTIR data for further machine learning. The gas chamber is sealed during the operation to improve the accuracy of the IPA concentration measurement. The oscilloscope is connected to the plate electrode collector to record the dark current response during the sliding of the multi-switched triboelectric nanogenerator.

### AI algorithms for IPA concentration estimation

Synthetic minority oversampling technique is a synthetic data algorithm with comprehensive sampling, which can be used to solve the problem of unbalanced data distribution. Owing to the limitation of data collection, it is difficult to ensure that the information of sampled IPA data in different concentrations is distributed evenly, yielding worse performance in the predictive model. Thus, SMOTE which realizes in MATLAB R2022a is adopted for each concentration of IPA data. The data of each concentration should be equal in amount so that it can be used in the classification or regression model. The deep neural network is developed for the gas feature regression and IPA concentration estimation. The proposed DNN is developed on python 3.8 using the Keras and sci-kit-learn packages. Our DNN model is specifically constructed by multiple connected layers: dense (50, “relu”) → dropout (0.2) → dense (15, “relu”) → dense (1, “linear”). The batch size is 20. The epoch number is 300. The loss function between the predict value and the true value is a mean-squared error. The optimizer is Adam. The ratio settings for train/test split are 70:30, 75:25, and 80:20. The result with different ratio settings are shown in Figs. S21–S23, where the increased training amount results in a higher accuracy. All data before regression or classification is preprocessed. The normalization is implemented and transformed the data range into [−1,1]. The asymmetric least squares calibration is applied for the baseline subtraction by the second derivative constrain weighted regression. The estimated mean error using improved IMMS technique is about 40.15 ppm~52.03 ppm.

### AI algorithms for IPA identification from gas mixture

The t-SNE is used in exploratory data analysis and for nonlinear dimensionality reduction. t-SNE reserves the local characteristics by transforming the data distance relationship into a probability distribution. When implementing t-SNE, the same amount of low-dimensional data is randomly generated, and then the loss function measures the difference between two probability distributions. The gradient descent method is used to update this batch of data, and the low-dimensional data satisfying the requirements is obtained. The t-SNE is performed in MATLAB R2022a to intuitively visualize the feature space in two and three dimensions. The support vector machines by the “libsvm” package in MATLAB 2022a are implemented to compare with LDA.

### Reporting summary

Further information on research design is available in the Nature Portfolio Reporting Summary linked to this article.

## Supplementary information

Supplementary Information

Reporting Summary

## Data Availability

All technical details for producing the figures are enclosed in Methods and Supplementary Information. Data are available from the corresponding author C.L., J.Z., and A.S. upon request.

## Code availability

The codes that support the findings of this study are available from the corresponding author C.L., J.Z., and A.S. upon request.
