# Supplementary material for: Triboelectric-induced ion mobility for artificial intelligence-enhanced mid-infrared gas spectroscopy
Source: Nat Commun. 2023 May 2;14:2524. doi: 10.1038/s41467-023-38200-6 (PMC10154418; doi:10.1038/s41467-023-38200-6)
Supplement: Supplementary file 1 — Supplementary Information [file 41467_2023_38200_MOESM1_ESM.pdf]

Supplementary Information

Triboelectric-induced ion mobility for artificial intelligence-enhanced mid-infrared gas spectroscopy

Jianxiong Zhu[1,*], Shanling Ji[1], Zhihao Ren[2,3,4], Wenyu Wu[1], Zhihao Zhang[1], Zhonghua Ni[1], Lei Liu[1], Zhisheng Zhang[1], Aiguo Song[5,*], Chengkuo Lee[2,3,4,*]

[1]School of Mechanical Engineering, Southeast University, Nanjing, 211189, P. R. China

[2]Department of Electrical and Computer Engineering, National University of Singapore, Singapore, 117576, Singapore

[3]Center for Intelligent Sensors and MEMS (CISM), National University of Singapore, Singapore, 117576, Singapore

[4]NUS Suzhou Research Institute (NUSRI), Suzhou, 215123, P. R. China

[5]School of Instrument Science and Engineering, Southeast University, Nanjing, 211189, P. R. China

Correspondence and requests for materials should be addressed to C. Lee (email: elelc@nus.edu.sg), J. Zhu (email: mezhujx@seu.edu.cn), A. Song (email: 101005200@seu.edu.cn)

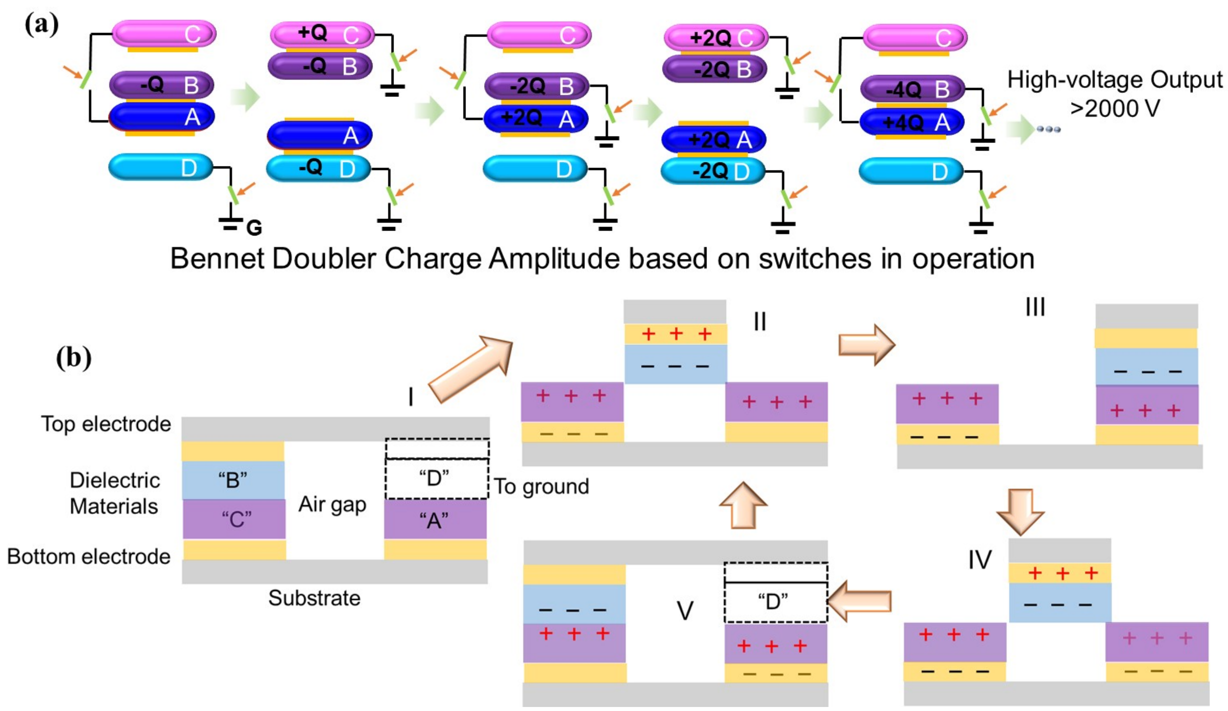

Figure S1 Mechanics of the high-voltage generator. (**a**) Mechanism of Bennet doubler in operation, and (**b**) the designed multi-switched manipulation triboelectric nanogenerator from Bennet doubler-inspired.

The mechanics of the high-voltage generator is from the operations of multi-switched manipulation ("on" or "off") in time sequence. Both triboelectric effect and electrostatic induction generate initial charges onto dielectric "B" "C" and "D" "A", and the electrode at the backside of dielectric "B" will be charged with the same quantity but opposite charges. Due to the charges transfer in "C" and "D", the multi-switches will operate between "on" and "off" action, resulting in a continuous charge accumulation onto the electrode "A". Thus, the charges manipulation in time sequence would convert the mechanical energy to electric energy by leveraging the accumulated charge onto dielectric "A" and dielectric "B" from the ground.

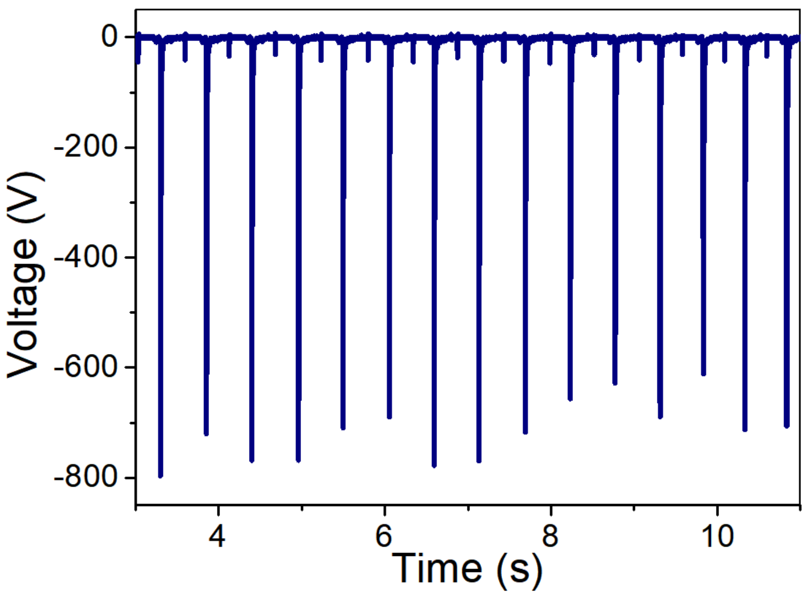

Figure S2 Output voltage from the device by oscilloscope.

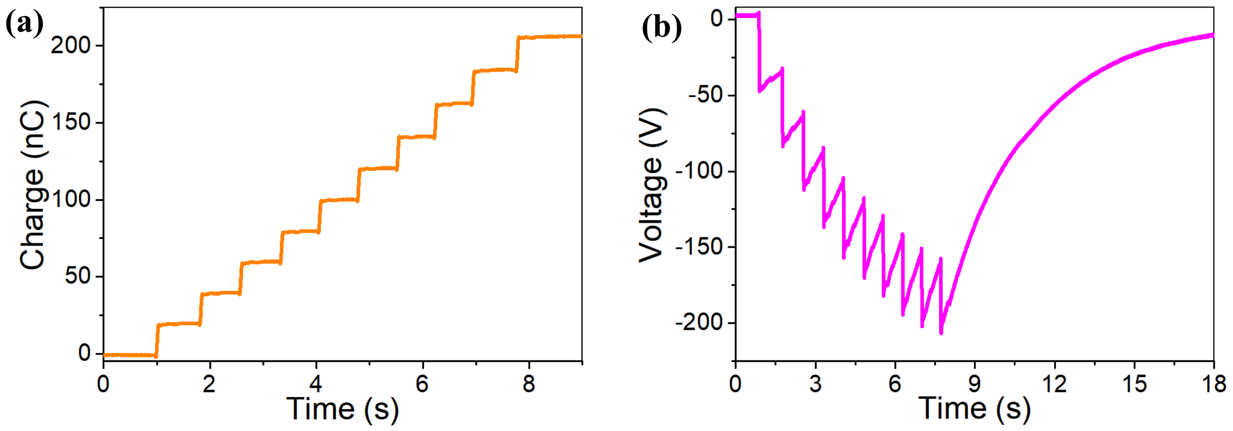

Figure S3 Obtained parameters values from triboelectric generator device. (**a**) Q_{oc} from PTFE vs FEP, and (**b**) V_{oc}.

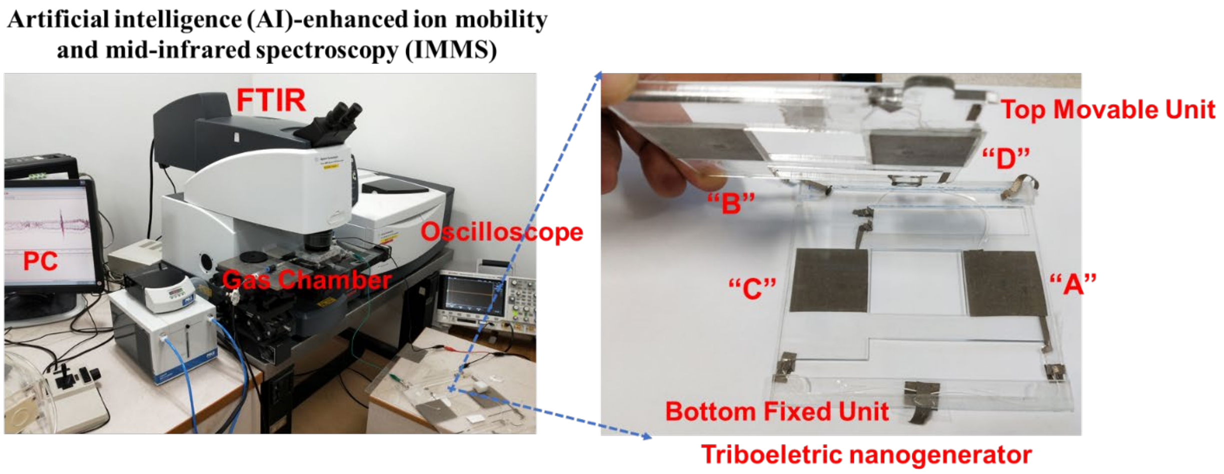

Figure S4 The artificial intelligence-enhanced ion mobility and mid-infrared spectroscopy, its multi-switched manipulation triboelectric nanogenerator, and the artificial intelligence program.

The software of our AI-enhanced system is based on the Pycharm 2022.2.3 (Community Edition). The detail code about the concentration prediction is as follows,

```
from keras.models import Sequential
from keras.layers import Dense,Dropout
import numpy as np
import pandas as pd
import time
from sklearn.model_selection import train_test_split
from sklearn.preprocessing import StandardScaler
scaler=StandardScaler()
##########################Load Dataset#############################
ipa_data=np.genfromtxt("D:/.../IPA.csv",delimiter=',')

# X_samp and y_samp contain the oversampled dataset
print(np.shape(ipa_data))
len_para=len(ipa_data[0,:])
X=scaler.fit_transform(ipa_data[:,:len_para-1])
y=ipa_data[:,-1]
y=scaler.fit_transform(y.reshape(-1,1))
x_train,x_test,y_train,y_test=train_test_split(X, y,test_size=0.3)
print(np.shape(x_train))
##########################metircs error###############################
def regression_evaluation(true_value: np.ndarray, pred_value: np.ndarray) -> pd.DataFrame:
    #:param true_value:
```

```
    #:param pred_value:
    from sklearn.metrics import mean_squared_error
    from sklearn.metrics import mean_absolute_error
    from sklearn.metrics import r2_score
    r2 = r2_score(true_value, pred_value)
    mae = mean_absolute_error(true_value, pred_value)
    rmse = np.sqrt(mean_squared_error(true_value, pred_value))
    mse = mean_squared_error(true_value, pred_value)
    smape = 100 / len(true_value) * np.sum(
        2 * np.abs(pred_value - true_value) / (np.abs(true_value) + np.abs(pred_value)))
    print(f"MLP MAE test: {mae}")
    print(f"MLP MSE test: {smape }")
    print(f"MLP r2 test: {r2 }")

    dataframe = pd.DataFrame([r2, mae, rmse, mse, smape]).T
    dataframe.columns = ['r2', 'MAE', 'RMSE', 'MSE', "SMAPE"]
    return dataframe
####################DNN##################################
# net model
model = Sequential()
model.add(Dense(units = 50,
                activation='relu',
                input_shape=(x_train.shape[1],)
               ))
model.add(Dropout(0.2))
model.add(Dense(units = 15,activation='relu' ))
model.add(Dense(units = 1,activation='linear'  ))

print(model.summary())

model.compile(loss='mse',optimizer='adam',)
start_time=time.time()
history = model.fit(x_train, y_train,
              epochs=300,
              batch_size=20,
              verbose=0,
              validation_data = (x_test, y_test)
             )
end_time=time.time()
print("Time: {:.2f}s".format(end_time - start_time))
import matplotlib.pyplot as plt
plt.plot(history.history['loss'])
plt.plot(history.history['val_loss'])
plt.title('Model loss')
plt.ylabel('Loss')
plt.xlabel('Epoch')
```

```
plt.legend(['Train', 'Test'], loc='upper left')
plt.show()
#Test
start_time=time.time()
y_mlp_pred = model.predict(x_test)
end_time=time.time()
print("Time: {:.2f}s".format(end_time - start_time))
y_mlp_pred = scaler.inverse_transform(y_mlp_pred)
y_test=scaler.inverse_transform(y_test)
#Metrics
vv=regression_evaluation(y_test,y_mlp_pred)
#Plot figures
plt.plot(y_test,'*--',color='cornflowerblue',label='Target (ppm)')
plt.plot(y_mlp_pred,'o-',color='blue',label='Prediction (ppm)')
plt.xlabel('Sample number',fontdict={'family' : 'Arial', 'size': 15})
plt.ylabel('Gas concentration (ppm)', fontdict={'family': 'Arial', 'size': 15})
plt.legend(fontsize=10,loc="upper right")
plt.tick_params(labelsize=10)
plt.show()
np.save('test_or.npy',y_test)
np.save('pred_or.npy',y_mlp_pred)
```

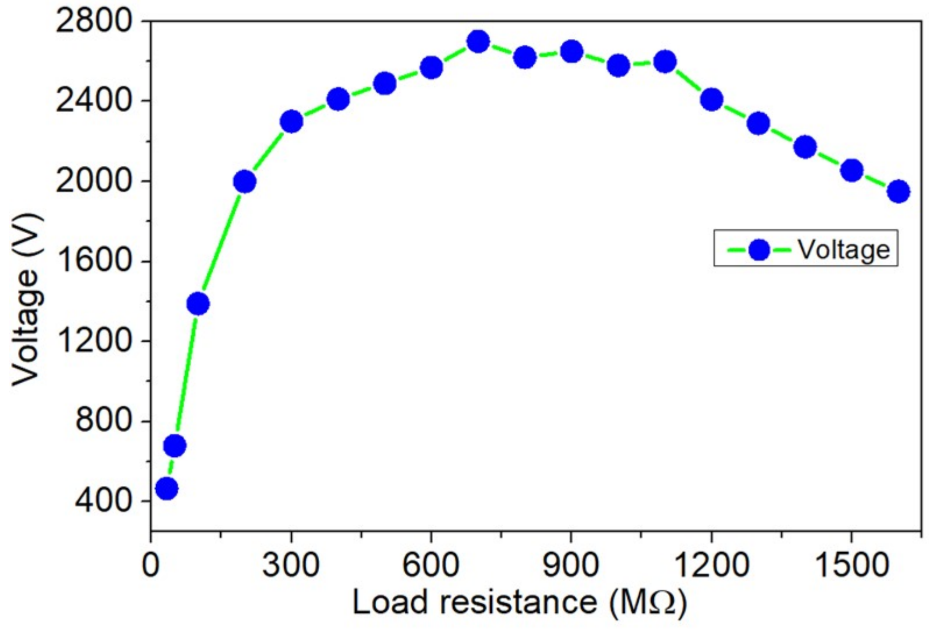

Figure S5 Load resistance vs voltage was obtained from our group in Bennet doubler-inspired triboelectric nanognerator [1-2] to support the triboelectric device in system.

References

[1] Zhu J, Sun Z, Xu J, Walczak R, Dziuban J, Lee C. Volatile organic compounds sensing based on bennet doubler-inspired triboelectric nanogenerator and machine learning-assisted ion mobility analysis. Science Bulletin, 2021, 66, 1176-1185.

[2] Zhu J, Ren Z, Lee C. Toward healthcare diagnoses by machine learning-enabled volatile organic compound identification. ACS Nano, 2021, 15, 1, 894–903.

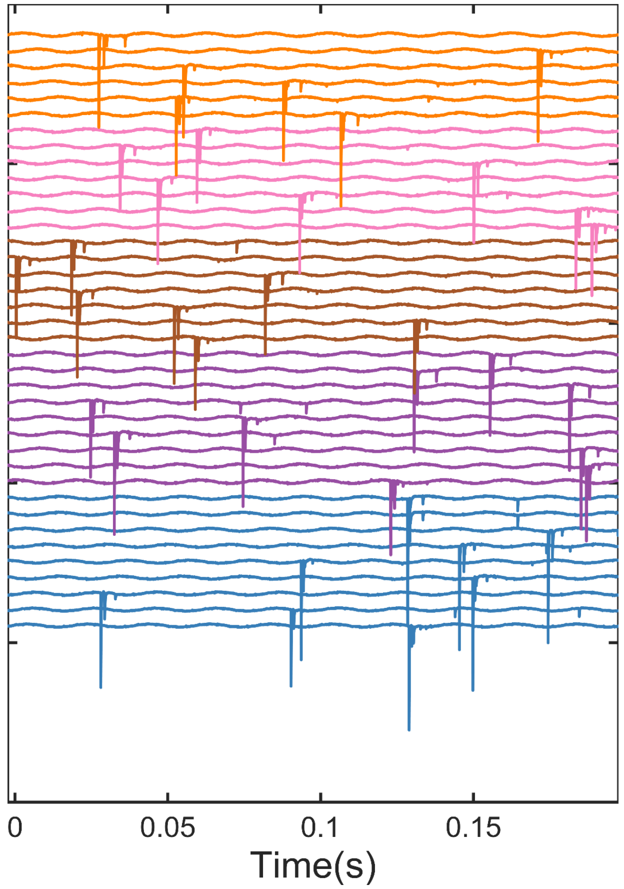

Figure S6 The raw data from ion mobility.

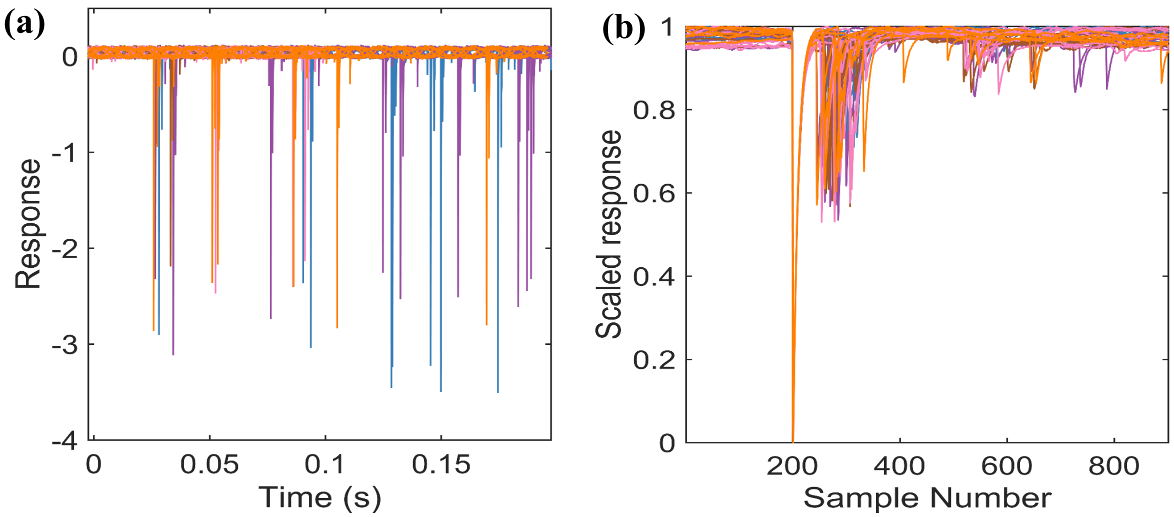

Figure S7 Ion mobility data. (**a**) Raw data from ion mobility with a uniform, and (**b**) peak calibration for ion mobility.

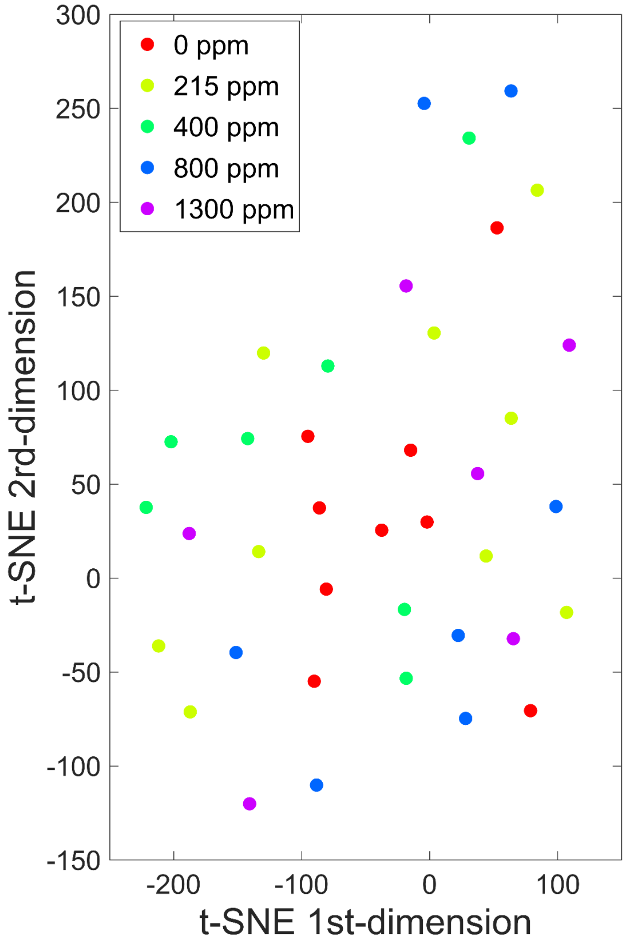

Figure S8 t-SNE extracted features of raw data from ion mobility.

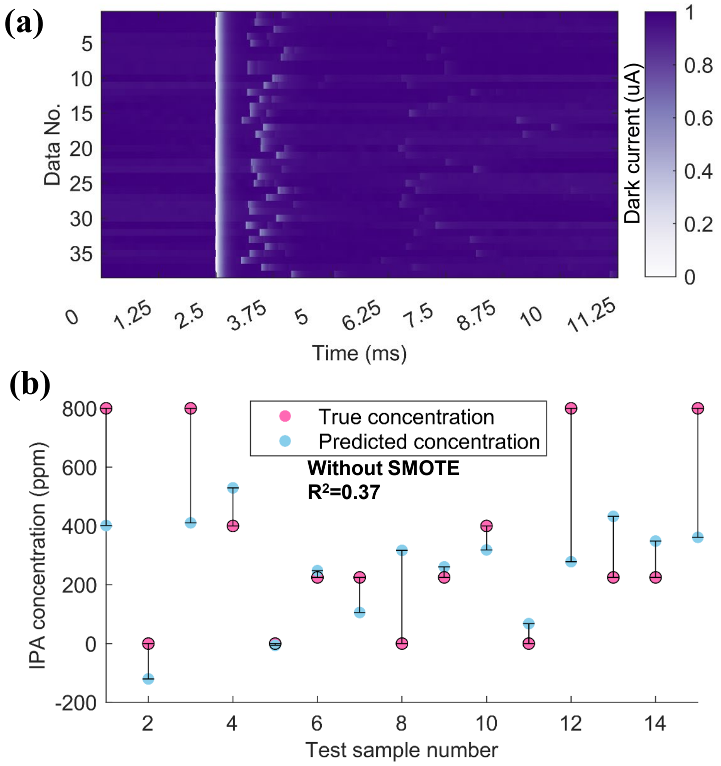

Figure S9 SMOTE+DNN for ion mobility. (**a**) Raw data of ion mobility in data map. (**b**) Ion mobility without SMOTE+DNN.

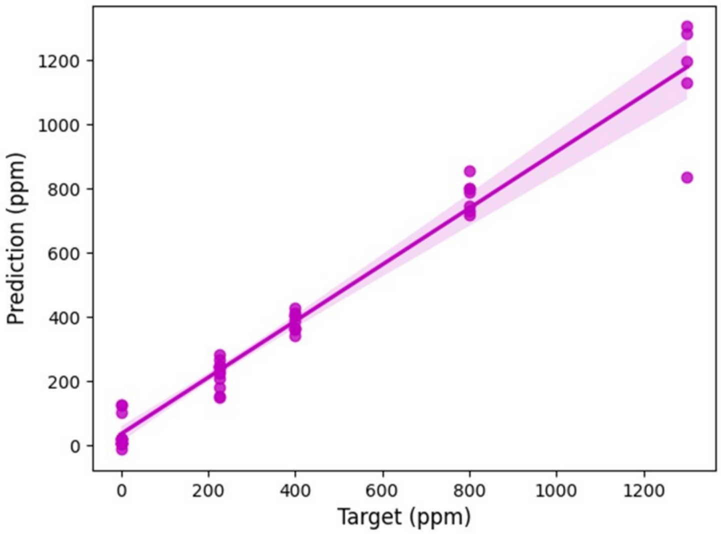

Figure S10 Results of prediction for ion mobility.

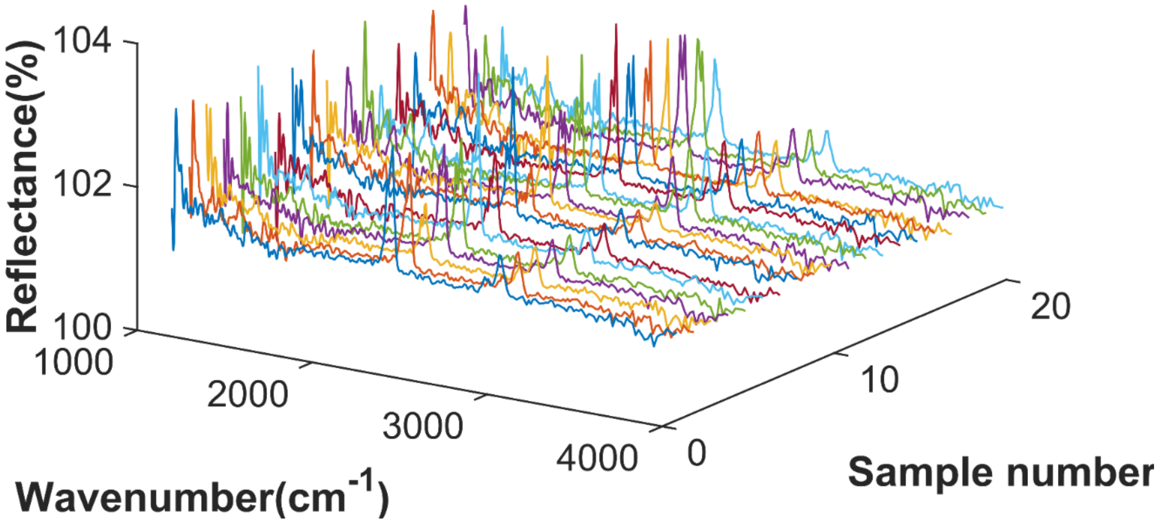

Figure S11 The mid-infrared spectrum with the time slot.

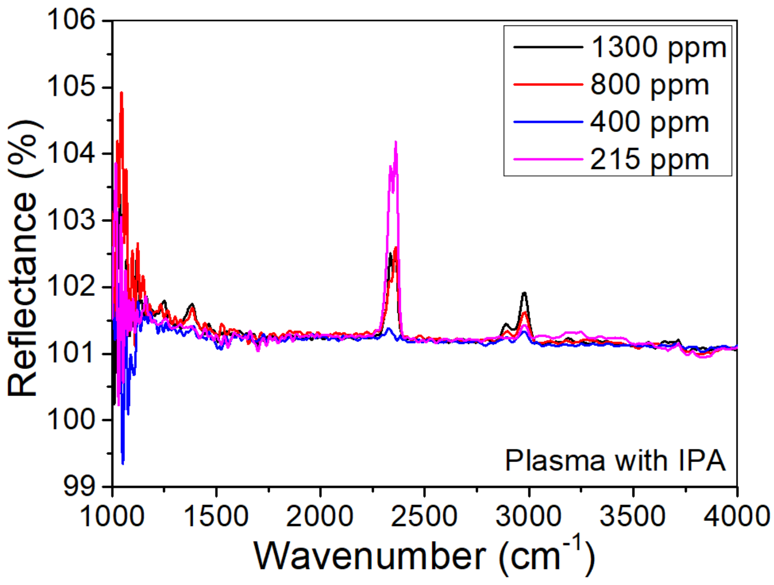

Figure S12 The range scale of the response of the mid-infrared of different concentrations in IPA.

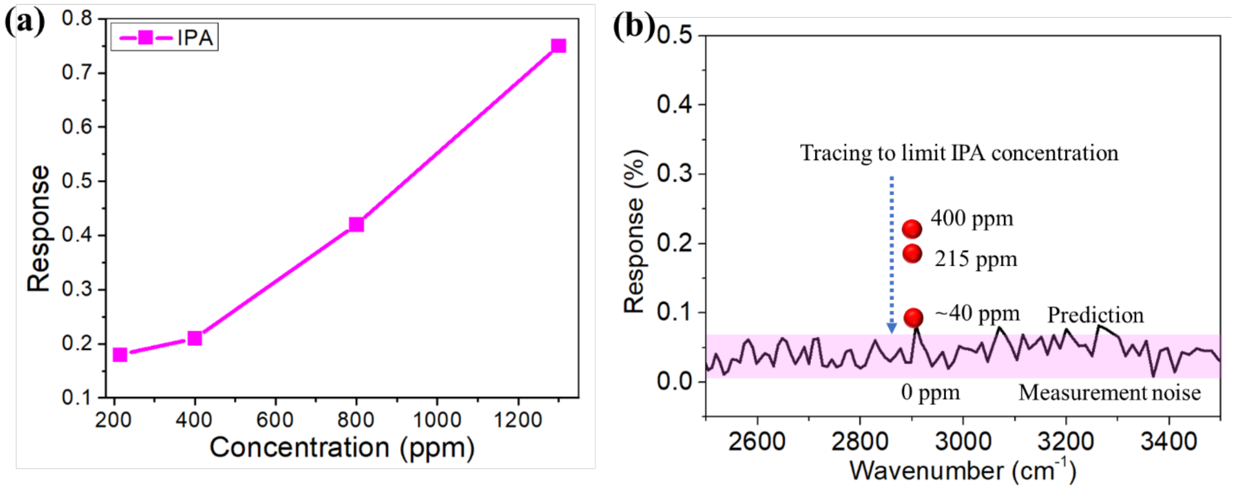

Figure S13 Response and limit of detection (LOD). (**a**) The relationship of the peak response of the mid-infrared to different concentrations in IPA, and (**b**) LOD of the system for IPA detection.

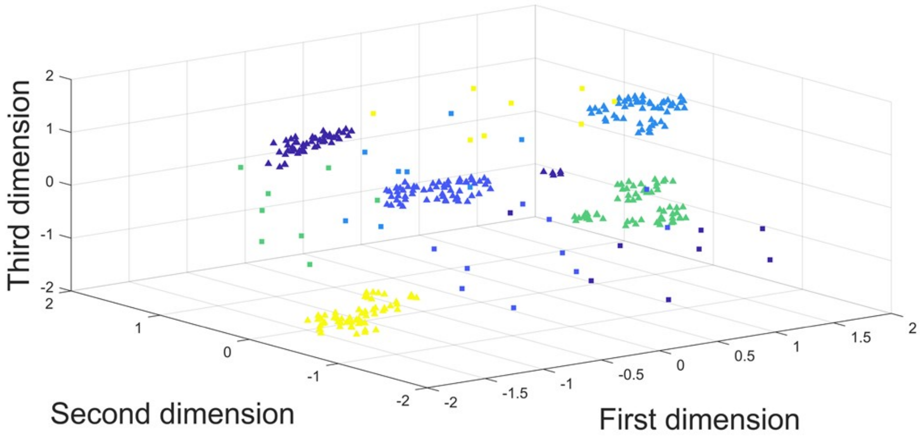

Figure S14 3D dimension of mid-infrared.

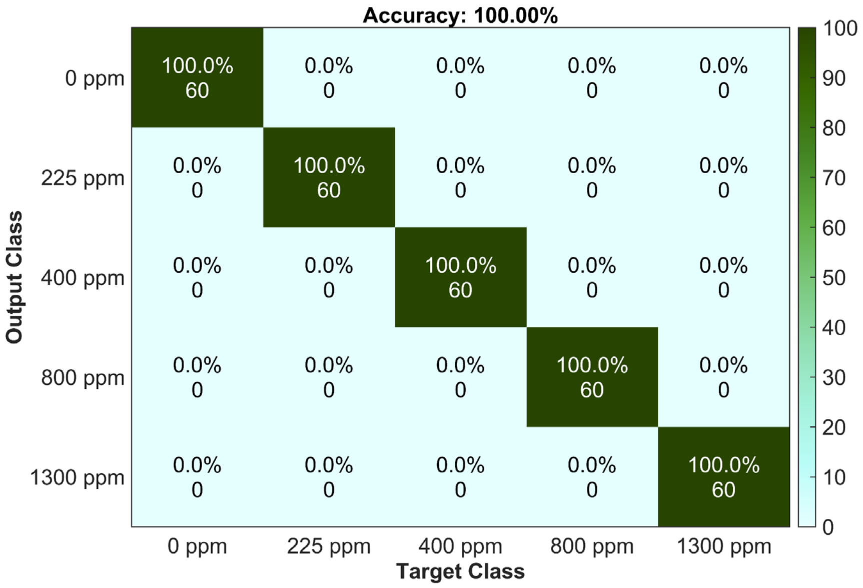

Figure S15 Accuracy of mid-infrared.

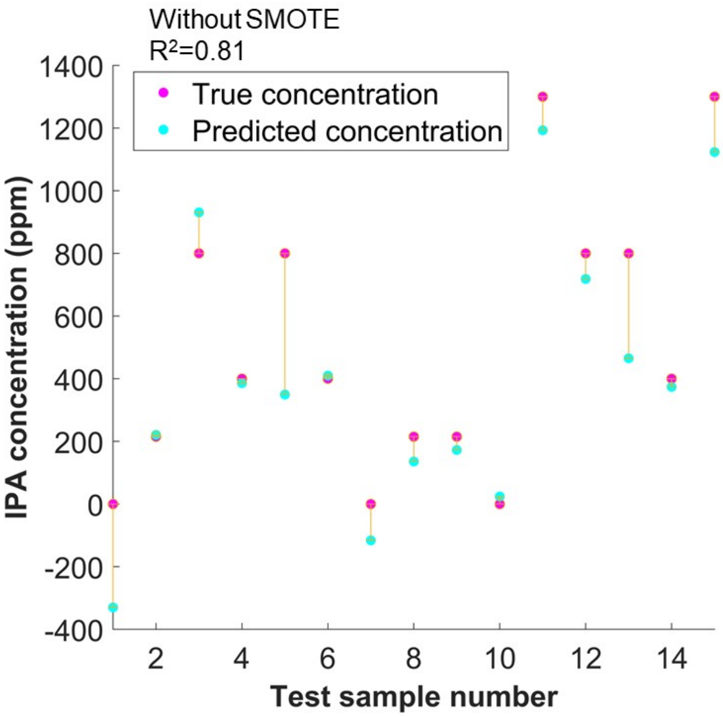

Figure S16 Results without SMOTE+DNN augmentation in R^2*=081*.

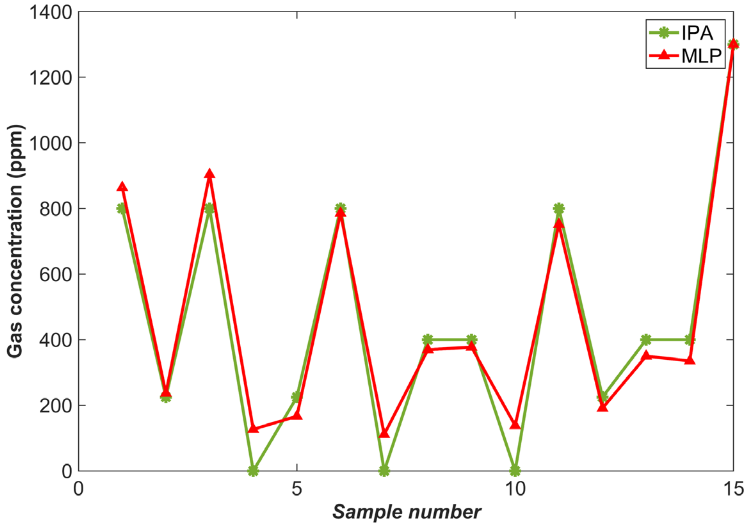

Figure S17 Results of SMOTE+DNN from R^2 score from 0.87 to 0.98.

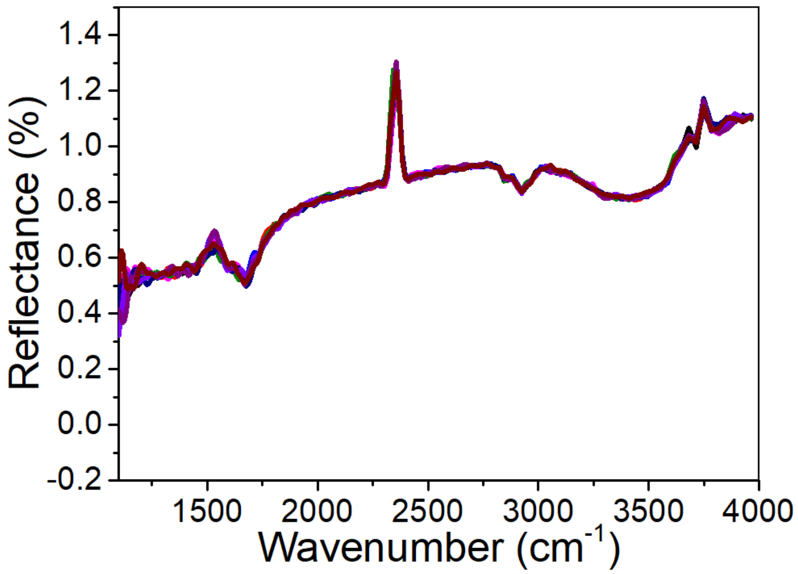

Figure S18 Without background calibration of the FTIR.

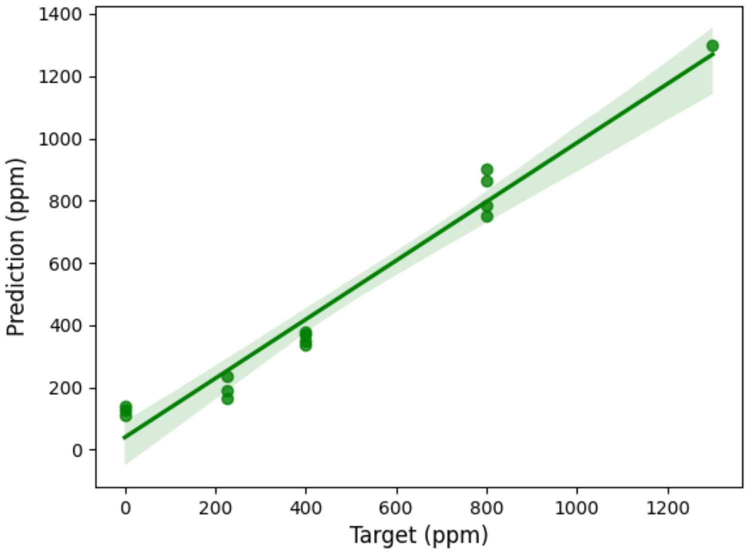

Figure S19 Results of the case about background calibration of the FTIR.

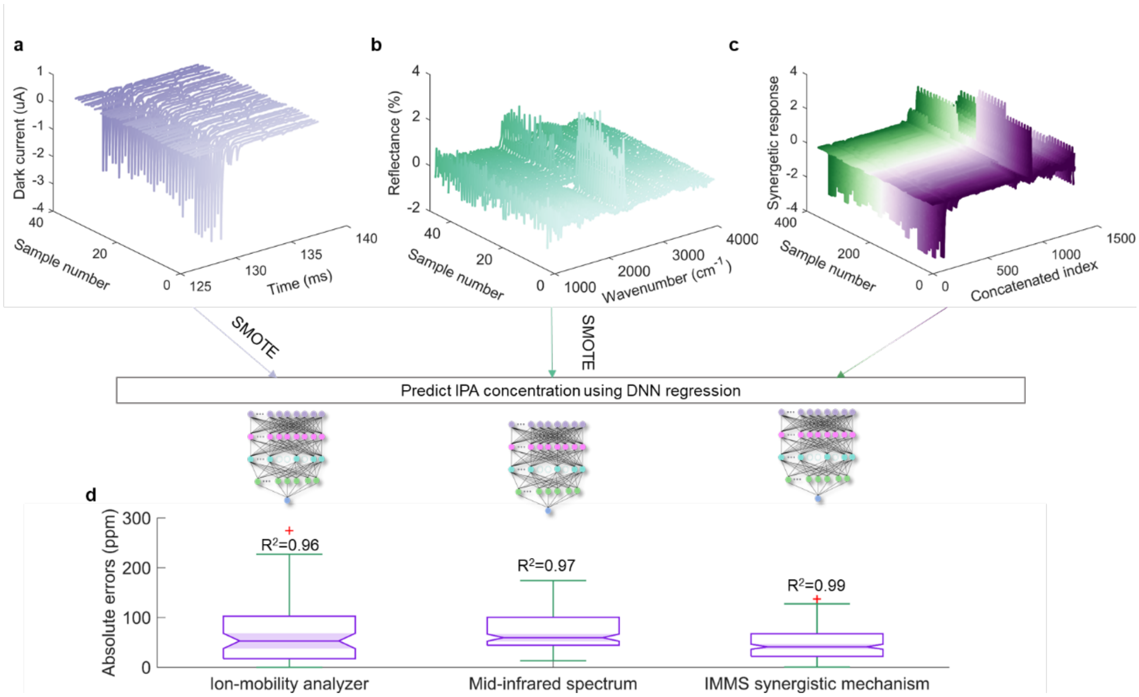

Figure S20 AI-enhanced the synergistic methodology for the ion-mobility system, mid-infrared system, and IMMS. (**a**) The ion-mobility raw data for different IPA concentrations. (**b**) The mid-infrared raw data for different IPA concentrations. (**c**) The synergistic data by concatenation. (**d**) The prediction of absolute errors in the IPA concentration estimation by SMOTE+DNN regression.

Since the SMOTE is proved to be helpful in the characteristics with a small amount of raw data, the ion-mobility data (Figure S20a) and plasma-enhanced mid-infrared data (Figure S20b) are extended by SMOTE. Although the two generations are not carried out at the same time, the concatenation operation still compensates for the underlying information between ion mobility and mid-infrared sensing, as shown in Figure S20c. The same DNN regression model is utilized to estimate IPA concentration by taking the aforementioned data. The prediction performance is summarized in Figure S20d. The compensatory information heightened the AI model performance in the IPA concentration prediction, wherein the overall distribution of absolute

errors is lower. The R^2 score using the synergetic mechanism achieves 0.99 while that using single sensing data is 0.96~0.97.

Table S1 Response performance in IPA concentration estimation

	Ion-mobility analyzer	Mid-infrared spectrum	IMMS synergistic mechanism
Training number	107	210	266
Training time	7.48 s	8.73 s	10.19 s
Testing number	45	90	114
Testing time	0.12 s	0.14 s	0.15 s
Characteristics	Lower estimation accuracy; Simpler calculation	Lower estimation accuracy; Simpler calculation	Wider range concentration detection; Higher Accuracy

The time costs for training among ion-mobility analyzer, mid-infrared spectrum, and IMMS synergistic mechanism ranged from 7.48 s to 10.19 s, while their training number is 107, 210, and 266. On the other hand, the testing time is less than 0.15 s, even for the 114 testing data from IMMS synergistic mechanism.

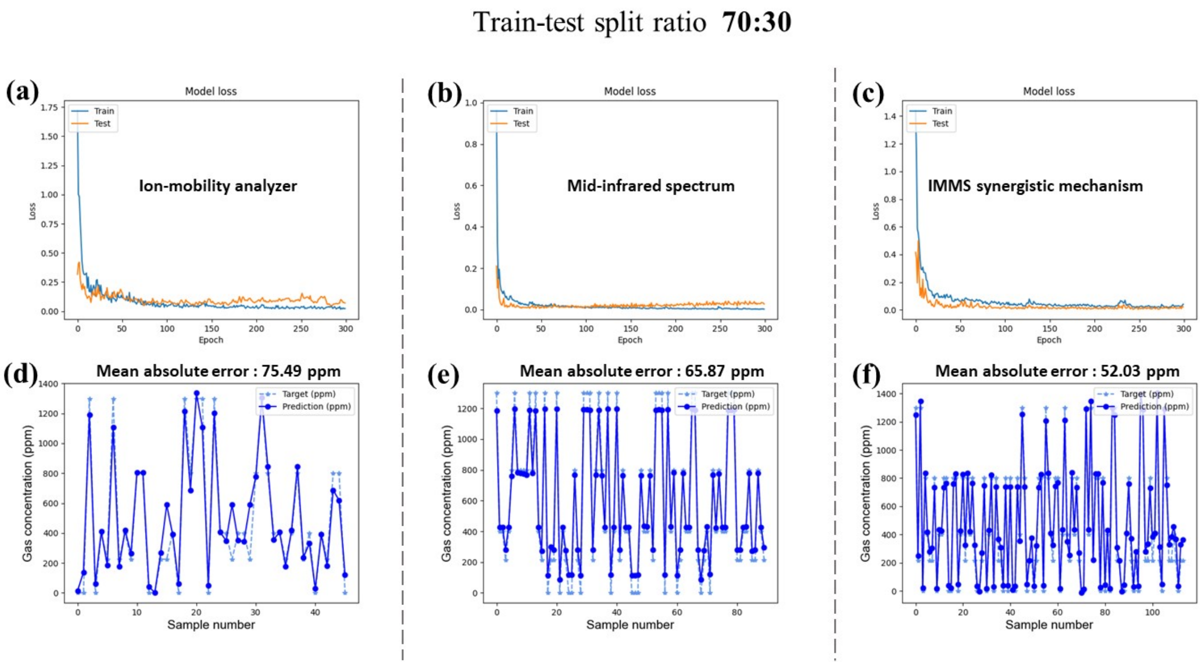

Figure S21 IPA concentration estimation using ion-mobility analyzer (**a**) and (**d**), mid-infrared spectrum (**b**) and (**e**), and IMMS synergistic mechanism (**c**) and (**f**). The train-test split ratio is 70:30.

Train-test split ratio **75:25**

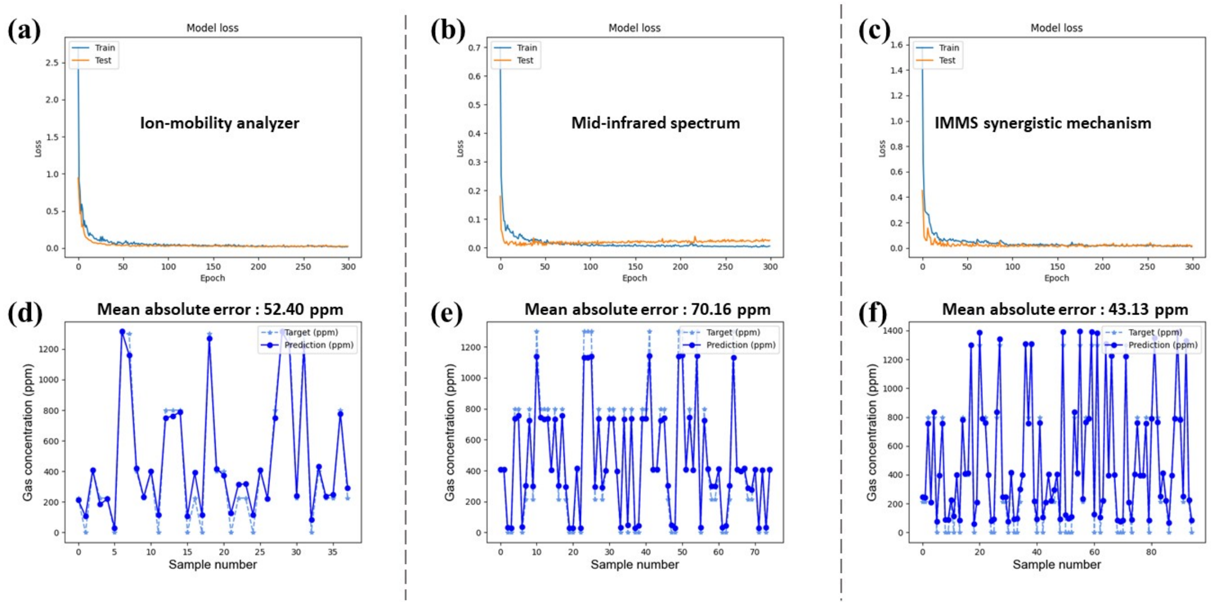

Figure S22 IPA concentration estimation using ion-mobility analyzer (**a**) and (**d**), mid-infrared spectrum (**b**) and (**e**), and IMMS synergistic mechanism (**c**) and (**f**). The train-test split ratio is 75:25.

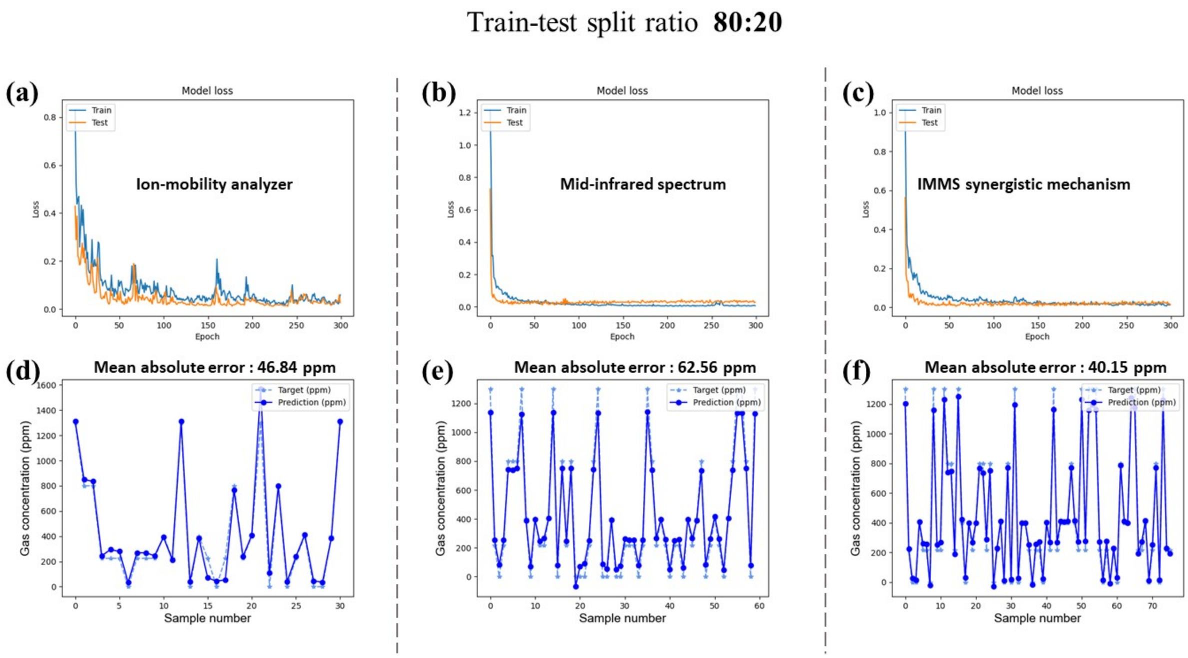

Figure S23 IPA concentration estimation using ion-mobility analyzer (**a**) and (**d**), mid-infrared spectrum (**b**) and (**e**), and IMMS synergistic mechanism (**c**) and (**f**). The train-test split ratio is 80:20.

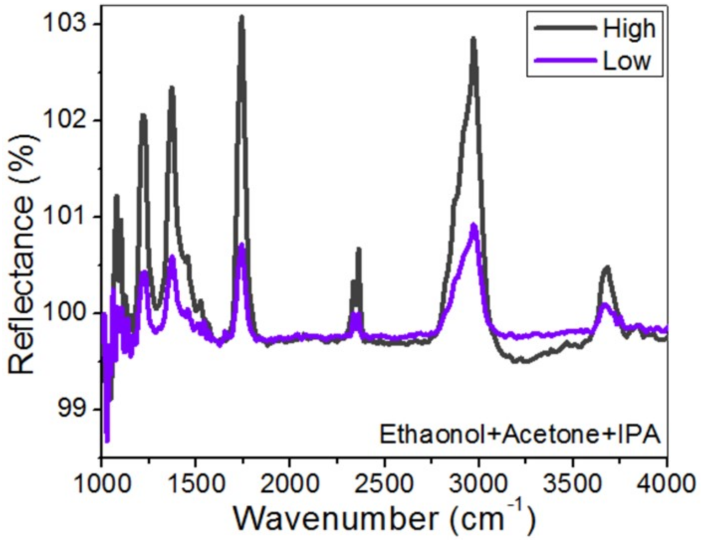

Figure S24 Mid-infrared raw data with a different mixture IPA, three mixture gases.

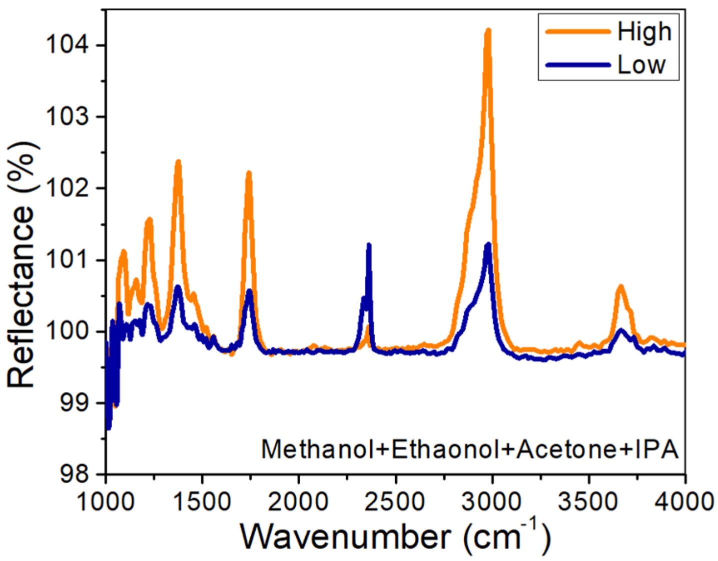

Figure S25 Mid-infrared raw data with a different mixture IPA, four mixture gases.

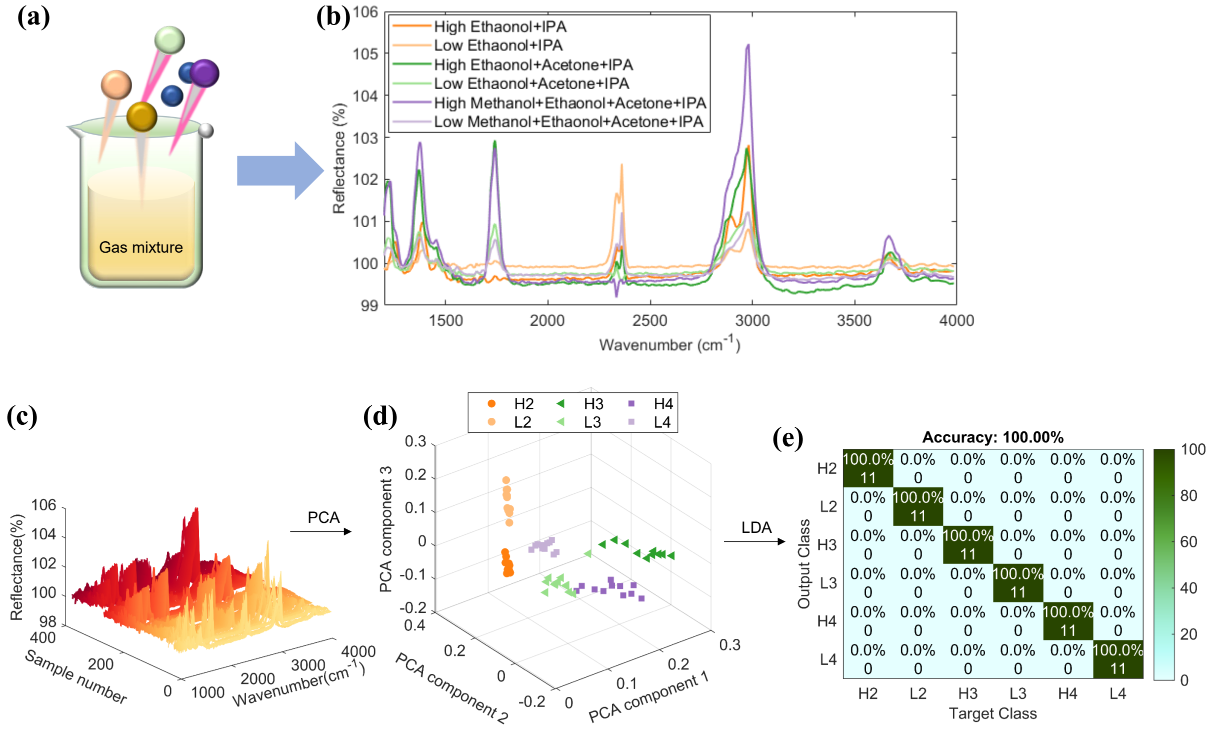

Figure S26 Mixture gas detection based on the plasma-enhanced mid-infrared spectrum. **(a)** Gas mixture for measurement. **(b)** The mid-infrared characteristics with a different mixture IPA. **(c)** Mid-infrared raw data. **(d)** PCA feature to all the mixture IPA. **(e)** AI-enhanced method for mixture gas identification with 100% accuracy.

The IPA molecules in the IMMS approach are dramatically shaken by the super-high voltage. The data of the gas mixture (Figure S26a) is addressed such that the mixture characters are classified using PCA and LDA. As shown in Figure S26b, IPA mixed with one ("H2" and "L2"), two ("H3" and "L3"), or three ("H4" and "L4") different kinds of species appear. A higher IPA concentration in the IPA gas mixture induces a strong peak response at a wavenumber of ~ 3000 cm^{-1}, clearly implying that the peak response of different gas species can be well-identified. Assisted by the PCA and LDA methods, the raw mid-infrared generation can be classified as shown in Figure S26c-e.

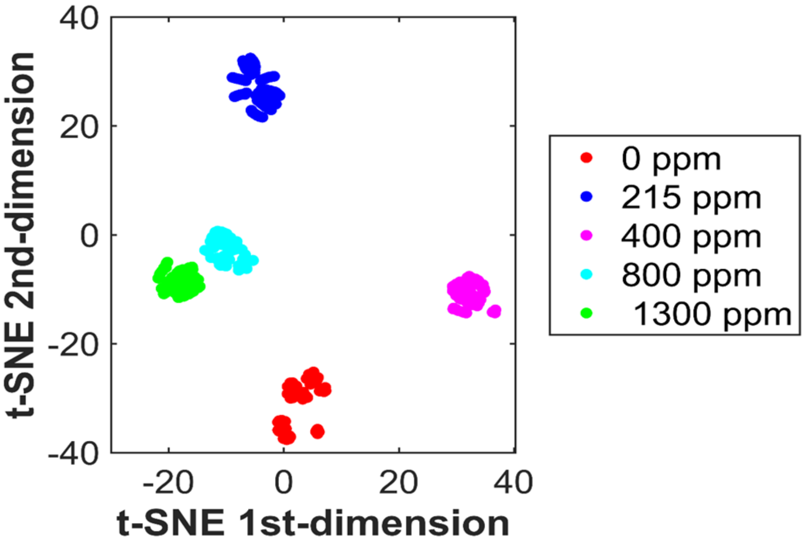

Figure S27 t-SNE feature to all the different IPA concentrations.
